# Optimizing the development and evaluation of complex interventions: lessons learned from the BetterBirth Program and associated trial

**DOI:** 10.1186/s43058-020-00014-8

**Published:** 2020-02-25

**Authors:** Dale A. Barnhart, Katherine E. A. Semrau, Corwin M. Zigler, Rose L. Molina, Megan Marx Delaney, Lisa R. Hirschhorn, Donna Spiegelman

**Affiliations:** 1 Harvard T.H. Chan School of Public Health, Boston, MA USA; 2 Ariadne Labs, Boston, MA USA; 3 Brigham and Women’s Hospital, Boston, MA USA; 4 Harvard Medical School, Boston, MA USA; 5 University of Texas, Austin, TX USA; 6 Dell Medical School, Austin, TX USA; 7 Beth Israel Deaconess Medical Center, Boston, MA USA; 8 Northwestern University Feinberg School of Medicine, Chicago, IL USA; 9 Center for Methods in Implementation and Prevention Science and Department of Biostatistics, Yale School of Public Health, New Haven, CT USA

**Keywords:** Complex intervention, Intervention development, India, Childbirth, WHO Safe Childbirth Checklist

## Abstract

**Background:**

Despite extensive efforts to develop and refine intervention packages, complex interventions often fail to produce the desired health impacts in full-scale evaluations. A recent example of this phenomenon is BetterBirth, a complex intervention designed to implement the World Health Organization’s Safe Childbirth Checklist and improve maternal and neonatal health. Using data from the BetterBirth Program and its associated trial as a case study, we identified lessons to assist in the development and evaluation of future complex interventions.

**Methods:**

BetterBirth was refined across three sequential development phases prior to being tested in a matched-pair, cluster randomized trial in Uttar Pradesh, India. We reviewed published and internal materials from all three development phases to identify barriers hindering the identification of an optimal intervention package and identified corresponding lessons learned. For each lesson, we describe its importance and provide an example motivated by the BetterBirth Program’s development to illustrate how it could be applied to future studies.

**Results:**

We identified three lessons: (1) develop a robust theory of change (TOC); (2) define optimization outcomes, which are used to assess the effectiveness of the intervention across development phases, and corresponding criteria for success, which determine whether the intervention has been sufficiently optimized to warrant full-scale evaluation; and (3) create and capture variation in the implementation intensity of components. When applying these lessons to the BetterBirth intervention, we demonstrate how a TOC could have promoted more complete data collection. We propose an optimization outcome and related criteria for success and illustrate how they could have resulted in additional development phases prior to the full-scale trial. Finally, we show how variation in components’ implementation intensities could have been used to identify effective intervention components.

**Conclusion:**

These lessons learned can be applied during both early and advanced stages of complex intervention development and evaluation. By using examples from a real-world study to demonstrate the relevance of these lessons and illustrating how they can be applied in practice, we hope to encourage future researchers to collect and analyze data in a way that promotes more effective complex intervention development and evaluation.

**Trial registration:**

ClinicalTrials.gov, NCT02148952; registered on May 29, 2014

Contributions to the literature

- Complex interventions often fail to produce the desired results in full-scale trials. However, there is little guidance on how to best develop complex interventions, especially in early stages of the intervention development process.
- We identified lessons learned from the BetterBirth Program, a complex intervention designed to implement the World Health Organization’s Safe Childbirth Checklist in India, and provide illustrative examples showing how these lessons can be applied in practice.
- This study complements existing literature on complex intervention development by highlighting the importance of producing a robust theory of change and demonstrating how quantitative analysis can be used to refine theories of change and promote the development of more effective complex interventions.

## Background

Complex interventions consist of a package of several interacting components or implementation strategies [1–3] and are widely used in public health including HIV prevention [4], smoking cessation [5], and childhood obesity prevention [6]. Complex interventions are ideally both effective, or able to produce a heath impact, and optimized, or able to efficiently use available resources in a way that produces the greatest impact possible. Currently, there is little consensus on how to best develop complex interventions. Methodologically rigorous approaches, such as the factorial or fractional-factorial designs often used in the Multiphase Optimization Strategy (MOST), can estimate causal effects of individual package components [7, 8]. However, these designs require researchers to specify detailed information on candidate components at the beginning of the study, which may not be feasible early in the development process. They can also be prohibitive in cluster randomized studies where few units are available for randomization [9] or the cost per unique treatment condition is high [10]. The recently developed Learn-as-You-Go (LAGO) design allows researchers to estimate the effects of individual components using data collected in phases, with data from previous phases being used to recommend interventions for subsequent phases [11]. However, this design has yet to be used in a real-world study.

In practice, complex interventions are often developed and refined using qualitative research and expert and stakeholder consensus [12–15]. These approaches are rarely accompanied by quantitative analyses estimating the effectiveness of individual implementation components or demonstrating that the intervention has been sufficiently optimized to warrant a full-scale evaluation. Consequently, many interventions fail to produce desired impacts on health outcomes in a full-scale trial [16]. One recent example of this phenomenon is the BetterBirth intervention, a complex intervention designed to improve the quality of care in childbirth facilities with the goal of improving maternal and neonatal health. Despite extensive preliminary research during the intervention development process [17], the intervention did not improve maternal and newborn health in a recent high-profile trial, although it did improve birth attendant adherence to evidence-based practices [18]. This paper identifies lessons learned from the BetterBirth experience and provides illustrative examples showing how these lessons could be applied to the development and evaluation of future complex interventions.

## Methods

### Overview of the BetterBirth intervention

BetterBirth is a complex intervention consisting of a multi-component implementation package designed to promote the use of the World Health Organization’s Safe Childbirth Checklist. The 28-item checklist is intended to help birth attendants successfully complete evidence-based essential birth practices (EBPs) that focus on prevention and early identification of complications during facility-based deliveries [19]. BetterBirth was developed through a multi-phase process. The initial implementation package was informed by team members’ prior experiences successfully implementing a similar checklist-based quality improvement tool, the Safe Surgical Checklist [20, 21], and by a pilot study conducted in a hospital in Karnataka, India [22]. This initial package was refined over three sequential development phases conducted in primary-level health facilities in Uttar Pradesh, India. The first two phases, pilot 1 and pilot 2, were pre-post studies conducted in two and four facilities, respectively. The third development phase occurred among the first 15 control-intervention pairs enrolled in a matched-pair, cluster randomized trial (CRT) designed to assess the effectiveness of the BetterBirth intervention on reducing maternal morbidity and maternal and newborn mortality [17, 23]. We consider these 15 pairs to constitute a development phase because researchers originally planned to conduct preliminary analyses among these facilities and further adapt the intervention as needed prior to enrolling the remaining CRT facilities. However, time and budgetary constraints ultimately prevented further adaptations to the final implementation package. To accommodate both the pre-post and matched-pair, cluster randomized designs, we designate all births occurring in a control site or any births occurring in an intervention site prior to the introduction of the BetterBirth intervention as part of the “control period” and any birth occurring in an intervention site after the introduction of the BetterBirth intervention as part of the “intervention period.”

Across the three development phases, the content, delivery, and intensity of implementation package components assigned to facilities varied, as described by Hirschhorn et al. [17] and summarized in Table 1. During pilot 1, the BetterBirth intervention included three package components: leadership engagement, an educational and motivational program launch, and ongoing coaching visits to promote checklist use and EBP adherence. The fourth package component, a data feedback cycle in which birth attendants were provided with quantitative information on performance, was added to the pilot 2 and CRT phases. BetterBirth is considered a complex intervention because it uses these multiple, potentially interacting implementation strategies to promote birth attendant checklist use and behavior change. In each phase, the intervention’s effectiveness was assessed based on birth attendants’ checklist use and EBP adherence, which was directly observed by trained independent nurses and recorded using standardized data collection tools. EBP adherence data was collected during three distinct “pause points” during delivery: on admission to a facility, just before pushing, and within 1 h after birth. However, practical considerations related to the timing and duration of labor prevented all births from being continuously observed from admission through discharge such that not all EBPs were observed for each birth. Data on EBP adherence were available on 113 births from pilot 1, 2369 births from pilot 2, and 6562 births from the 15 pairs of sites from the CRT development phase.

**Table 1:** The BetterBirth implementation package by phase

Phase	Leadership engagement	Educational and motivational launch	Data feedback	Coaching visits
Pilot phase 1	Non-standard initial engagement, with a focus on facility rather than district leadership	3-day launch featuring 1 day of flipchart and video-based training, 1 day of checklist demonstrations and placement of checklist posters on walls, and 1 day of facilitated practice sessions on checklist use	None	1 coaching visit every 2 weeks for the first 6 months, then 1 coaching visit per month
Pilot phase 2	Standardized initial engagement with district and facility leadership	Semi-standardized 2-day launch featuring flipchart, videos, checklist posters, roleplaying, and the identification of a childbirth quality coordinator	Ongoing feedback, using paper-based reports. Frequency of report generation and delivery to sites unspecified	3 coaching visits per week for the first 4 weeks, then less frequently
Cluster randomized trial (CRT)	Standardized initial engagement with district and facility leadership. Semi-regular meetings with district leadership	Standardized 2-day launch featuring flipchart, videos, checklist posters, roleplaying, the identification of a childbirth quality coordinator, and a safe-childbirth pledge	Ongoing feedback using app-based reports. Frequency of report generation and frequency of sites reviewing feedback in the app are unspecified	2 coaching visits per week for months 1–4; 1 coaching visit per week for months 5–6; 1 coaching visit per fortnight in month 7; 1 coaching visit per month in month 8

### Identifying lessons learned

The intervention that was developed as a result of these three development phases was tested in a large-scale matched-pair, cluster randomized trial (CRT) designed to assess the effectiveness of the BetterBirth intervention on reducing maternal morbidity and maternal and newborn mortality [23]. The results of this trial showed that the intervention did not improve maternal and newborn health, although it did improve birth attendant adherence to evidence-based practices [18]. To identify barriers preventing the identification of an optimal BetterBirth implementation package, we reviewed published articles, research protocols, internal reports, data collection tools, implementation team weekly updates, and data from all three development phases of the BetterBirth intervention. The results of this review were used to identify barriers that hindered the identification of an optimal intervention package and corresponding lessons learned. For each lesson, we described its importance and used material motivated by the BetterBirth Program to illustrate how this lesson could be applied in practice. These illustrative examples are designed to aid in the development and evaluation of future complex interventions.

### Illustrative examples and analyses

The theory of change (TOC) proposed in this paper was retrospectively developed following a review of the study materials and refined through discussion with members from the BetterBirth team. To assess the intervention’s overall effectiveness, we used a generalized linear model adjusted for development phase (pilot 1, pilot 2, and CRT phases), the intervention (vs. control) period, and their interactions [24]. When assessing the effectiveness of coaching, we added coaching intensity to the model, calculated for each birth as the number of coaching visits occurring at their facility in the 30 days prior to their birth. In pilot 2, only the first and last dates of coaching and the total number of coaching visits per site were recorded, so we calculated coaching intensity metrics by imputing the missing coaching dates assuming a uniform distribution bounded by the first and last coaching dates. To account for facility-level clustering, all standard errors were estimated using the empirical variance with an exchangeable working covariance structure.

## Results

We identified three key lessons learned: (1) develop a robust theory of change; (2) define optimization outcomes, which are used to assess the effectiveness of the intervention across development phases, and corresponding criteria for success, which determine whether the intervention has been sufficiently optimized to warrant full-scale evaluation; and (3) create and capture variation in the implementation intensity of intervention components. For each lesson, we describe its importance, discuss how it applies to the BetterBirth Program, and provide an illustrative example.

### Lesson 1: Develop a robust theory of change

The term theory of change (TOC) was popularized by Carol Wiess to describe a tool that defines and expresses researchers’ underlying assumptions and hypotheses about the processes through which a complex intervention improves outcomes [25–27]. The assumptions and hypotheses encoded in a TOC can be informed by a wide range of generalized theories commonly used in implementation science [28] including the Theory of Planned Behavior [29] or the Theoretical Domains Framework [30]. However, TOCs differ from generalized theories because they describe causal relationships between variables in a way that is specific to both the intervention of interest and the context in which that intervention is being implemented [26, 27]. TOCs should include the complex intervention’s individual components, primary outcome, and any process outcomes hypothesized to be on the causal pathway between at least one intervention component and the primary outcome. Additionally, TOCs should contain information on contextual factors expected to modify the relationship between these variables. Although many researchers use the terms logic model and TOC interchangeably, TOCs necessarily include information about the assumed causal connections between variables while logic models often assume simplistic progressions between groups of variables, such as inputs, outputs, outcomes, and impacts (e.g., [31, 32]) without making their causal assumptions explicit [26, 27].

The causal assumptions encoded in TOCs provide a structure for identifying and addressing the challenging hallmarks of complex intervention research [1, 2]. By identifying which hypothesized causal links are thought to be of greatest importance to the intervention’s overall success (or, alternatively, are the subject of greatest uncertainty), TOCs help prioritize data collection and guide subsequent data analyses [2, 25, 26, 33]. Testing for the existence of causal links hypothesized in the TOC can help identify ineffective intervention components, highlight incorrect assumptions about the underlying mechanism of change or context in which the intervention is being implemented, and inform future adaptions to the intervention [26, 33–35]. TOCs can also be used to identify appropriate data sources and units of analysis for each variable and can highlight which data sources will need to be linked together for analysis [36, 37]. Finally, TOCs can strengthen collaborations between interdisciplinary team members who may not otherwise share common assumptions or vocabulary for describing the intervention [25, 26, 38].

### Lesson 1: Application to BetterBirth

**Fig. 1:**
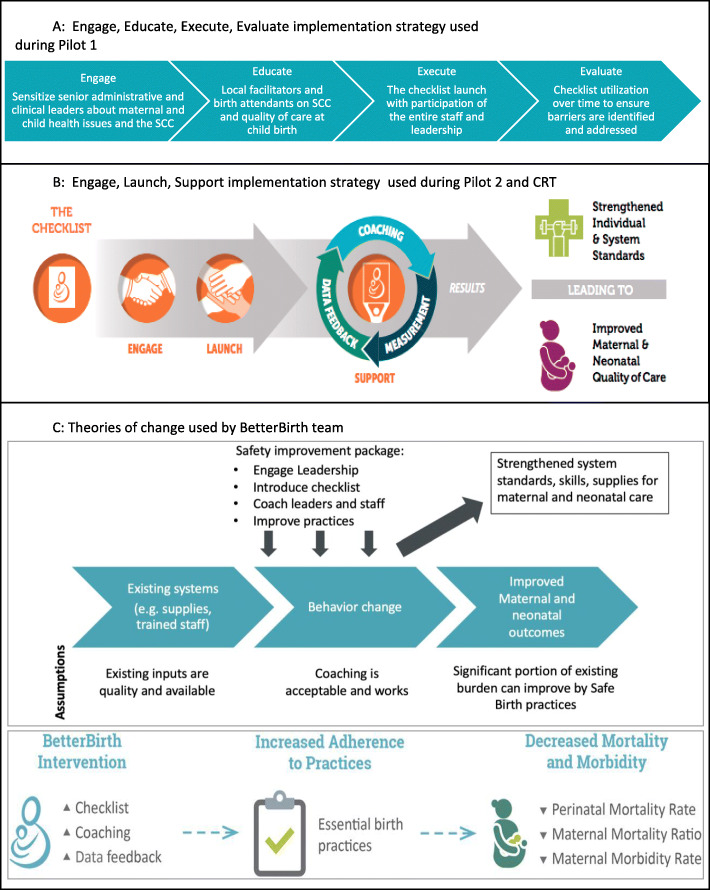
Implementation strategies and theories of change used during the development of the BetterBirth intervention

The BetterBirth team used two frameworks to describe their implementation strategies: the “Engage, Educate, Execute, and Evaluate” strategy in pilot 1 (Fig. 1a) and the “Engage, Launch, Support” strategy in the pilot 2 and CRT phases (Fig. 1b). Although these strategies were grounded in a generalized model known as the “4-Es” [39], they did not constitute a TOC because they lacked information on specific causal pathways through which individual implementation package components were hypothesized to improve maternal and newborn health. While these frameworks were effective at communicating the program’s overall implementation strategy, they could not be used to prioritize data collection, guide analyses, or suggest opportunities for adapting and improving the intervention. Subsequent theories of change that were developed were oversimplified given the intervention’s complexity and the context in which it was implemented (Fig. 1c), gaps which became most apparent during analysis [40].

**Fig. 2:**
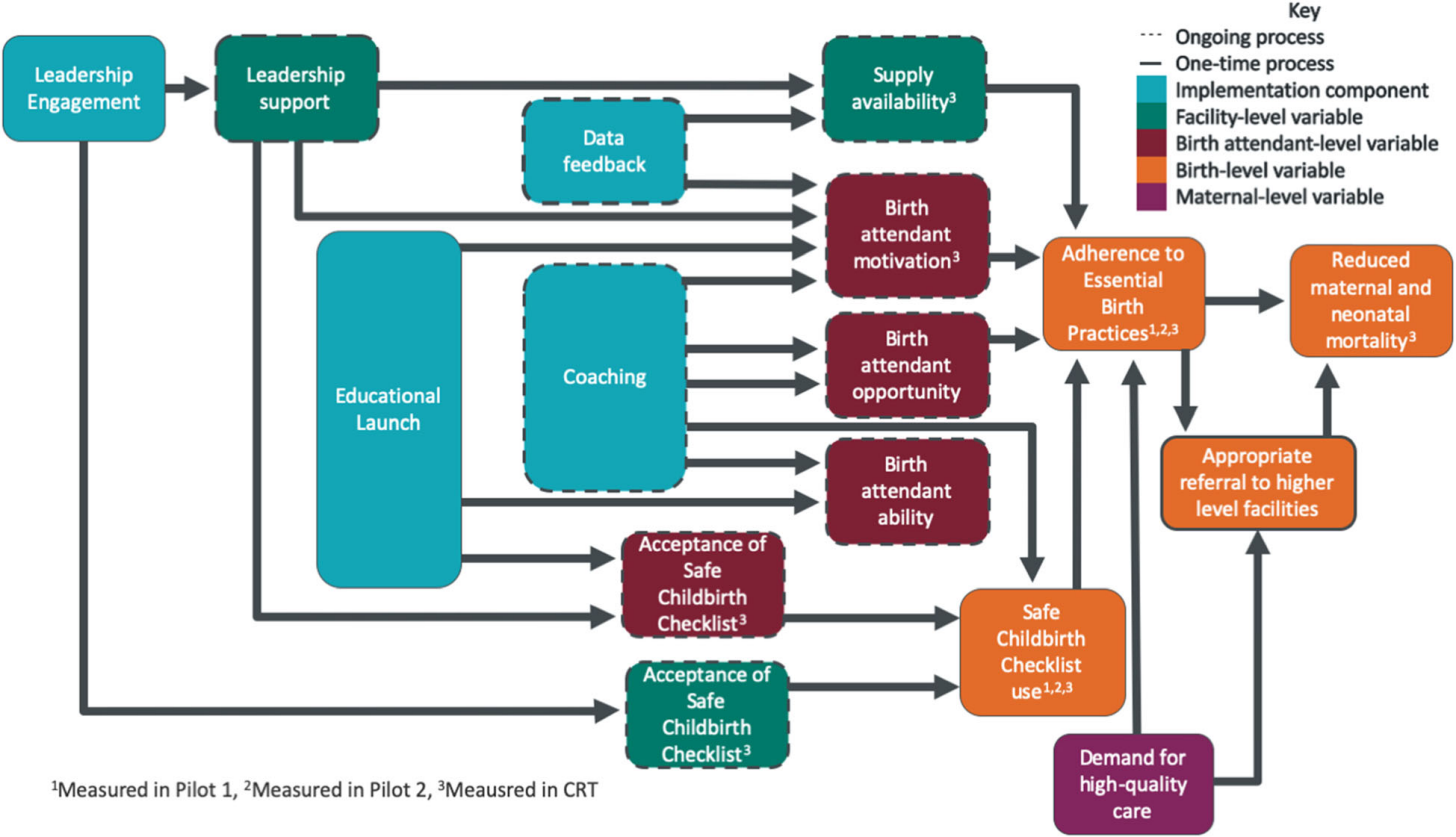
Robust theory of change for the BetterBirth intervention

We produced an alternative, robust TOC for the BetterBirth intervention (Fig. 2) using arrows to designate hypothesized causal relationships, shading to identify the desired unit of analysis for each variable, and superscripts to identify which variables were measured in each BetterBirth development phase. This retrospective TOC was developed primarily by program evaluators and implementors, but prospectively engaging community members and frontline healthcare providers can provide additional insight into local context and enhance community buy-in [41, 42]. Many variables identified in this TOC as playing important roles in the BetterBirth Program, such as birth attendant ability, were not measured during the development phases. Low baseline birth attendant ability has been hypothesized to be one factor that affected the overall success of the trial [18, 43, 44], and assessing this contextual factor earlier may have helped implementers address this barrier. Other variables, such as birth attendant attitudes towards the checklist, were measured in only a single phase and therefore could not be compared across phases, preventing deep understanding of how changes to the intervention affected these variables. Finally, the TOC contains hypothesized causal links that exist between variables that were assessed at different units of analysis. For example, attitude towards the checklist, which was assessed at the birth attendant level, was hypothesized to impact checklist use, which was assessed at the individual birth level. However, because the data collection process did not allow for individual birth attendants to be linked to individual births, this hypothesized link could only be assessed using data aggregated at the facility level. Developing a robust TOC at the start of the intervention development process could have highlighted these limitations, promoted more complete data collection, and provided additional opportunities to learn about the strengths and weakness of the intervention.

### Lesson 2: Select optimization outcomes and specify criteria for success

After creating a TOC, a subset of process outcomes can be selected as optimization outcomes. Optimization outcomes serve two functions during complex intervention development. First, comparing optimization outcomes across development phases identifies whether changes to the intervention have improved the intervention’s overall effectiveness. Second, comparing optimization outcomes against criteria for success identifies whether the complex intervention is sufficiently optimized to warrant evaluation in a full-scale trial. In the context of intervention development, criteria for success [45], which have also been described as optimization criteria [7] and “Go/No Go” rules [46], describe the minimal effect the intervention should have on optimization outcomes in order to produce a meaningful impact on the primary outcome. Interventions that fail to meet these criteria require additional phases of development before progressing to a full-scale evaluation.

To serve these two functions, optimization outcomes should be defined and assessed consistently across all development phases. They should also be valid surrogate outcomes for the primary outcome. Surrogate outcomes are sometimes used in clinical efficacy trials when collecting data on the primary outcome is expensive, time-consuming, or otherwise infeasible [46, 47]. If a surrogate outcome is valid, then the effect of the intervention on the surrogate outcome will correspond to the effect of the intervention on the primary outcome. However, it can be difficult to identify valid surrogate outcomes [48, 49]. Several high-profile trials have demonstrated that poorly chosen surrogates can lead to misleading conclusions about an intervention’s effectiveness [50–54]. Empirically validating surrogate outcomes requires data on intervention status, optimization outcomes, and primary outcomes [55], which are usually unavailable to researchers developing a new intervention. Without empirical verification of surrogate validity, researchers must rely on expert knowledge about the intervention and its expected effects to determine whether a candidate optimization outcome is likely to be a valid surrogate for the primary outcome.

In settings where the intervention is hypothesized to improve the primary outcome, researchers typically select an optimization outcome that (a) is positively correlated with the primary outcome and (b) is also expected to improve as a result of the intervention. In this setting, the following conditions will nearly always guarantee that the optimization outcome is a valid surrogate. More general conditions can be found elsewhere [48, 49]. First, the positive correlation between the surrogate and the primary outcome should reflect a positive causal effect and not be induced by confounding bias. For example, the BetterBirth team believed that increased checklist use would be positively correlated with maternal and newborn survival. However, checklist use would not have been a valid surrogate if this correlation was explained by bias, as would have occurred if more educated birth attendants were both more likely to use the checklist and more likely to have good patient outcomes. Second, if there are mechanisms through which the intervention could have unintended adverse effects on the primary outcome, those mechanisms should also adversely impact the surrogate outcome. For example, it is plausible that the BetterBirth intervention could have increased birth attendants’ adherence to specific tasks, such as providing oxytocin immediately after delivery, by decreasing the amount of time and resources they dedicated to other tasks, such as taking the temperature of newborns. In this case, adherence to any single EBP would have been unlikely to serve as a valid surrogate because it would not have reflected the potential unintended consequences of decreased adherence to other tasks. Third, if improvements in the surrogate are only beneficial among a specific subgroup of individuals, then the intervention should improve the surrogate outcome within that subgroup. For example, in the BetterBirth intervention, antibiotics were only expected to improve survival among the subgroup of women and newborns who were at risk of infection. Therefore, increases in antibiotic prescription rates would have only been a valid surrogate for survival if those increases occurred specifically among women and newborns identified as being at risk of infection.

In addition to selecting a valid optimization outcome, researchers must also specify criteria for success. Determining what criteria the optimization outcome must achieve for the intervention to have a reasonable chance of improving the primary health outcomes in a full-scale trial depends on both the strength of the relationship between the optimization outcome and the primary health outcomes and whether that relationship exhibits non-linear trends. This relationship can be relatively weak if the intervention does not target all major determinants of the primary health outcome. For example, in the BetterBirth Program, the association between EBP adherence and maternal and neonatal mortality may have been weaker than expected if key determinants of maternal and neonatal health that were not targeted by the intervention, such as inadequate antenatal care, were responsible for a substantial proportion of deaths. Situational considerations should also dictate whether criteria for success are set in relative or absolute terms. For example, if researchers believe that the optimization outcome must cross a certain threshold to impact the primary outcome, the criteria for success should reflect that absolute threshold, not relative improvements. If multiple process outcomes are selected as optimization outcomes, the criteria for success should account for each of these outcomes, either by combining them into a single composite outcome (e.g., average adherence across all EBPs must reach 86%) or by creating individual criterion for each outcome (e.g., adherence to each EBP much reach 70%). Selecting optimization outcomes and specifying criteria for success are both critical steps in the optimization process. If either is misspecified, then researchers could develop an intervention that improves the optimization outcome and meets the criteria for success but still fails to impact the primary outcome.

Finally, the sample size for each development phase should be calculated with respect to the optimization outcomes and their corresponding criteria for success. Phases do not necessarily need to be powered for formal hypothesis tests with a 5% type I error rate [46]. However, each phase should be powered such that estimates for the effect of the intervention on the optimization outcome are precise enough to inform a decision to proceed to the full-scale trial. For example, if the criteria for success are defined as observing a confidence interval for the optimization outcome that includes or exceeds some pre-specified value [56], then power calculations should ensure that the confidence intervals for the optimization outcome will be informatively narrow [45, 57].

### Lesson 2: Application to BetterBirth

Because the primary health outcomes of maternal morbidity and maternal and newborn mortality were relatively rare in the BetterBirth intervention’s small-scale development phases, adherence to individual EBPs was used to assess the intervention’s effectiveness. Adherence to each individual EBP was analyzed and reported independently, with different sets of EBPs used in different development phases [17, 18]. The criteria for success were not specified for any EBP. This approach had several limitations. First, adherence to a single EBP is unlikely to constitute a valid surrogate. As previously discussed, adherence to individual tasks would not capture potential unintended adverse consequences of the intervention. Furthermore, secondary analysis of BetterBirth Trial data suggests that individual EBPs were not correlated with improved health outcomes, suggesting that adherence to any individual EBP is not a valid surrogate for maternal and newborn health [58]. Second, without pre-defined criteria for success, it is unclear how EBP adherence data informed the decision to progress to a full-scale trial. Although the BetterBirth Trial observed large, statistically significant relative gains in EBP adherence, absolute adherence to EBPs remained low. For example, although intervention sites were 53 times more likely to use appropriate hand hygiene than control sites, they still used appropriate hand hygiene only 35% of the time [18]. If criteria for success had been defined in terms of absolute EBP adherence, these improvements may have been recognized as too modest to result in meaningful health improvements, triggering additional development phases. Third, these limitations were compounded by inconsistent data collection across the three development phases. Not all EBPs were assessed in all phases, and the timing and duration of EBP data collection relative to the start of coaching differed from phase to phase. Consequently, observed improvements in EBP adherence could have been caused either by real changes in the program’s effectiveness or by inconsistencies in the timing of data collection. Finally, while the CRT (*N* = 6562) was powered to detect 8.5 percentage point differences in EBP adherence between intervention and control sites, pilot 1 (*N* = 113) and pilot 2 (*N* = 2369) had relatively small sample sizes and were underpowered to provide meaningful estimates of EBP adherence.

Table 2 illustrates how a composite outcome of overall EBP adherence could have served as the optimization outcome and provided additional information about the intervention’s effectiveness prior to the full-scale trial. We defined overall EBP adherence as the proportion of observed EBPs that were successfully completed at each birth out of a set of eight EBPs that were measured consistently across all three phases (Table 2). This composite outcome was expected to serve as a valid surrogate for maternal and newborn survival for several reasons. First, overall EBP adherence was correlated with improved newborn survival [58], and it was assumed that this correlation reflected a causal effect. Second, the set of EBPs included in the composite outcome included a wide range EBPs which were measured at all three pause points and included EBPs performed on both women and newborns, so potential unintended adverse effects occurring at any stage in the birthing process would likely have been reflected by a reduction in overall EBP adherence. Finally, each EBP included in the composite outcome was believed to be beneficial for all births, not just for a certain subgroup. Our TOC supported the use of overall EBP adherence as an optimization outcome because (a) it was proximal to the primary outcome of newborn and maternal mortality and (b) there were no hypothesized causal pathways from the intervention components to newborn and maternal mortality that did not go through EBP adherence. We defined our criteria for success as observing an intervention period in which the 95% confidence interval for overall EBP adherence included 86%. This threshold was based on hypothesis that there may be a “threshold effect” in which most or all EBPs must be successfully completed to ensure comprehensive high-quality care for each woman and neonate and avoid potential adverse health events and on evidence from a previously successful implementation of the checklist in a hospital in Karnataka, India, where EBP adherence increased from 34 to 86% and researchers observed a marginally significant halving of stillbirths [22]. Our analysis suggested that, although the intervention increased EBP adherence in all phases, changes to the implementation package across phases did not meaningfully improve overall EBP adherence. Furthermore, baseline EBP adherence was low, and even though pilot 1 and pilot 2 were underpowered and had correspondingly wide confidence intervals, the 95% confidence intervals for overall EBP adherence during the intervention period did not include 86% in any phase. The intervention did not cross the threshold needed to achieve the criterion for success, suggesting that the implementation package was not sufficiently optimized to produce the desired health impact at the time of the trial.

**Table 2:** Effectiveness of each phase of the BetterBirth intervention on overall essential birth practice (EBP) adherence, which was calculated as the percentage of observed EBPs that were successfully completed out of eight EBPs consistently observed across all three phases: (1) use of a partograph, (2) maternal blood pressure at admission, (3) maternal temperature at admission, (4) appropriate hand hygiene prior to a push, (5) provision of oxytocin to the mother within 1 min of delivery, (6) assessment of baby weight, (7) assessment of newborn temperature, and (8) initiation of breastfeeding within 1 h. *N* = 9044 observations

Phase	Percentage point change in EBP adherence between intervention and control periods	Total EPB adherence during the intervention period
Pilot 1	9.7% (− 11%, 30%)	40% (23%, 56%)
Pilot 2	23% (17%, 28%)	37% (28%, 46%)
CRT^a^	33% (25%, 41%)	44% (39%, 50%)

^a^EBP adherence during CRT differs from what has been previously reported due to inclusion of 8, rather than 18, EBPs and because data is reported for entire post-intervention period rather than only for 2-month post-intervention and 12-month post-intervention periods

### Lesson 3: Create and capture variation in implementation intensity of components

If the criteria for success are not satisfied, investigating relationships between individual implementation components and other variables in the TOC can help identify strategies for improving the intervention. These analyses require researchers to first identify the distinct components and implementation strategies that make up their complex intervention and then to assess their variation in implementation intensity, which can be viewed as the “strength” or the “dose” of each intervention component. Implementation intensity can be quantified using domains that include content, quality, frequency, and duration [59, 60]. For each component, implementation intensity can be coded as a categorical variable (e.g., existence of data feedback system vs. no data feedback) or as a continuous variable (e.g., total number of data feedback reports provided). Variation in implementation intensity can arise from both planned and unplanned factors. Planned variation occurs when researchers assign study participants to receive different intensities of an intervention component, as is the case in multi-arm studies [61] and factorial designs [8]. Planned variation can also occur when researchers phase in, phase out, or otherwise change components’ intensities over time [59], as in a stepped wedge design [62] or when researchers adapt the intervention across sequential phases of development. Unplanned variation in implementation intensity is often described in terms of fidelity, or the extent to which the delivered intervention deviates from what was originally planned [63–65]. Although sources of variation in implementation intensity may be unplanned, they can be anticipated and measured. For example, researchers can document the dates of implementation component delivery; identify to whom components were delivered and to whom they were not; and use self-reported or expert reviews to assess whether the intervention’s delivery intervention occurred as planned [66].

Observing an association between the intensity of an individual implementation component and relevant process outcomes identified in the TOC can provide evidence to evaluate the effectiveness of that component [2]. As with all observational research, the extent to which this association reflects causal effects depends on the extent to which other confounders are accounted for [67–69]. In the case of complex interventions, special care should be taken to adjust for the remaining intervention components using either randomization (e.g., [70]) or analytic approaches (e.g., [71–74]). Researchers seeking to identify the effects of individual components should consider the extent to which various components’ implementation intensities are correlated with each other. The more strongly two components are correlated, the more difficult it is to identify their independent effects. Strong correlations often arise from the study design. For example, intractable collinearity occurs when researchers simultaneously introduce, intensify, or diminish the intensity of multiple components in a single arm or phase of a study. Collinearity can also occur if a common factor, such as highly motivated leadership, simultaneously affects fidelity to multiple intervention components. To better estimate the effectiveness of individual implementation components, researchers may wish to both create planned, uncorrelated variation in implementation intensity while also capturing unplanned variation that arises in the field.

### Lesson 3: Application to BetterBirth

Although the intensity of implementation package components varied across the BetterBirth development phases by design (Table 1), multiple components were simultaneously intensified in each phase. This practice created strong collinearities between individual components and prevented the identification of their individual effects. For example, the effect of having non-standardized leadership engagement could not be isolated from the effect of a 3-day launch duration since each of these conditions appeared only in pilot 1. Fidelity was not systematically measured for any component. However, the BetterBirth intervention did generate planned variation in the intensity of coaching, which occurred frequently in the intervention’s initial weeks’ intervention and became less frequent over time. In addition to this planned source of variation, the BetterBirth team gathered data on the dates of the coaching visits, allowing us to assess unplanned variation that occurred when sites deviated from the intended coaching schedule. Unfortunately, due to the multi-collinearity of the remaining components, coaching is the only intervention package component whose individual effect can be validly analyzed.

**Fig. 3:**
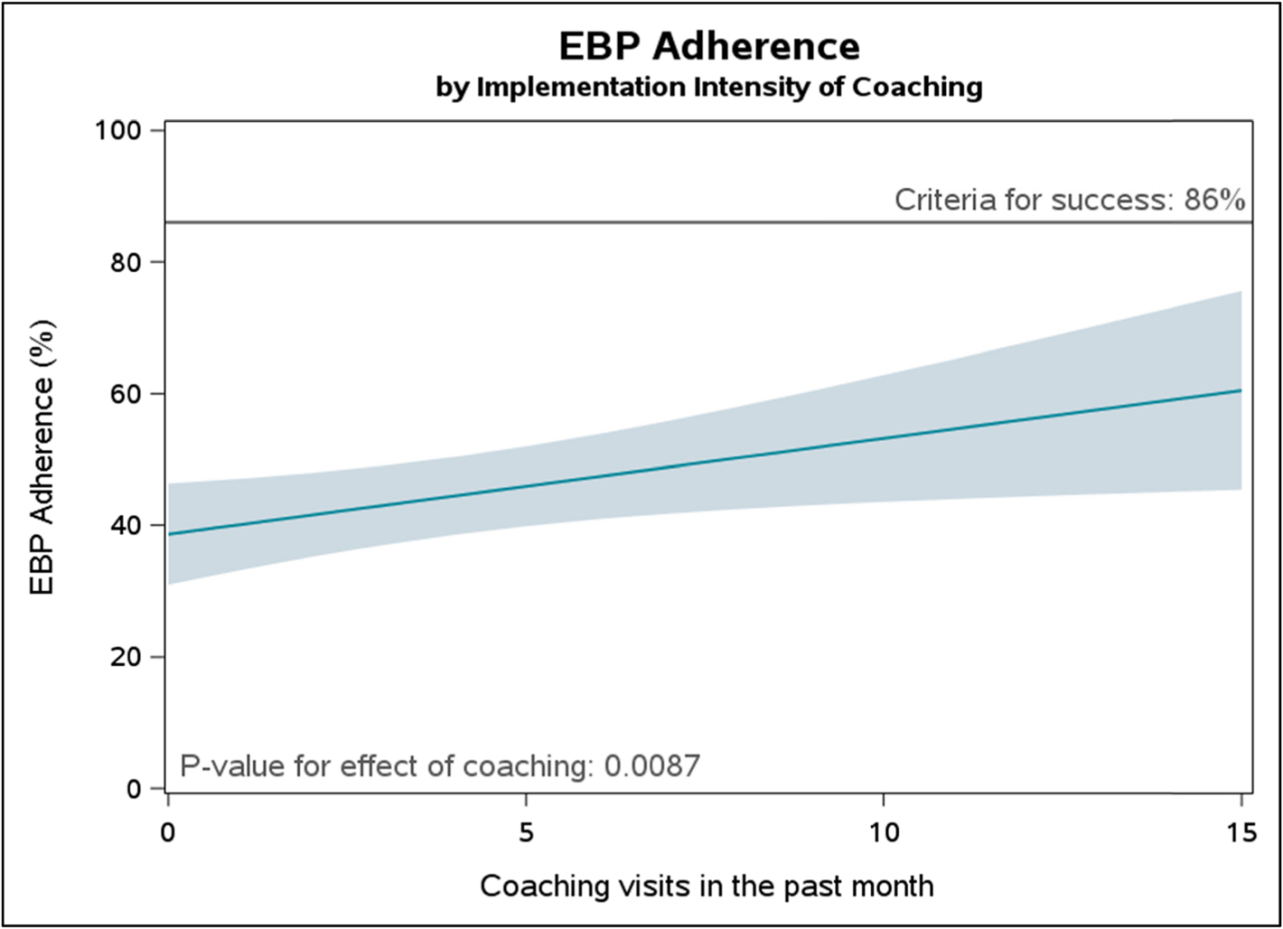
Dose-response relationship between coaching intensity and EBP adherence

In our TOC, we hypothesized that coaching would lead to increased EBP adherence. We tested for the existence of this relationship by assessing the association between coaching intensity, defined for each infant as the number of coaching visits occurring at their facility in the 30 days prior to their birth, and overall EBP adherence. We observed a linear dose-response relationship between the number of coaching visits per month and overall EBP adherence (Fig. 3). This association suggested that coaching was an effective intervention component. However, the model also illustrates that even 15 coaching visits per month would not have been sufficient to reach the criteria for success of 86% EBP adherence. Other implementation components may have needed to be added or intensified for the BetterBirth intervention to be effective.

## Discussion

Through our review of the BetterBirth intervention and associated trial, we identified three lessons learned that can help future researchers develop and evaluate complex interventions: (1) develop a robust theory of change, (2) define optimization outcomes and criteria for success, and (3) create and capture variation in the implementation intensity of individual components. Our illustrative examples demonstrate how these lessons could have been applied to BetterBirth. Specifying a TOC prior to data collection could have improved researchers’ understanding of the local context, promoted more complete data collection, and generated additional opportunities to learn about the intervention. Identifying an optimization outcome that was a valid surrogate for maternal and newborn health and comparing it against pre-defined criteria for success could have led to additional phases of intervention development prior to the full-scale trial. Finally, capturing and creating variation in implementation intensity for each implementation component could have helped identify which implementation components were effective and which needed additional adaptation.

These three lessons can be applied in both exploratory and methodologically rigorous phases of complex intervention development and evaluation. These lessons are highly compatible with the MOST framework, which also encourages researchers to begin with a well-developed theoretical framework and to only proceed to a full-scale trial after reaching some minimal effectiveness threshold [7, 10]. They are also compatible with the LAGO design, which relies on variation in implementation intensity to estimate the effect of individual components and optimizes the intervention relative to some pre-specified criteria for success [11]. While these lessons could be used to optimize interventions that are tested in a pure effectiveness trial, the theory of change, optimization outcomes, and implementation intensity metrics developed during the intervention optimization process could also be used to inform the design of full-scale evaluations that use a hybrid effectiveness-implementation design [75]. Although we intend for these lessons to apply to the development and evaluations of complex interventions, many recommendations, including carefully selecting surrogate outcomes, defining criteria for success, and adequately powering preliminary research, apply more generally to pilot and preliminary studies, as has been previously noted [45, 46, 57].

We recommend that researchers operationalizing these lessons consult previous recommendations for developing theories of change [26, 27] and assessing fidelity [63, 65, 66]. Surrogate outcomes have not been widely discussed in implementation science literature, so researchers may consult the epidemiologic literature for guidance [48]. While researchers cannot know a priori whether an optimization outcome is a valid surrogate for their primary outcome, using subject matter knowledge to critically evaluate the optimization outcomes’ role in the context of the intervention can promote the selection of valid optimization outcomes [76]. We anticipate that complex interventions will typically be optimized on process outcomes, rather the primary outcome itself, because development phases are usually too small or brief to collect sufficient data on the primary outcome. However, directly optimizing the intervention on the primary outcome would avoid the risk of selecting an invalid optimization outcome or setting inappropriate criteria for success.

The selection of the BetterBirth intervention as a case study is both a strength and limitation of this paper. During the BetterBirth Trial, the intervention was implemented with good fidelity [18] and the Safe Childbirth Checklist has been used successfully to improve clinical practice and health outcomes in other contexts [22, 77–82]. Therefore, the absence of an effect on the primary health outcomes in the BetterBirth Trial suggests that the intervention may not have been sufficiently optimized to address the specific contextual barriers to maternal and neonatal health in Uttar Pradesh [83]. Because the BetterBirth Trial was a high-profile, large-scale study conducted by a team with previous experience implementing behavioral change interventions [20, 21, 84, 85], it provides a realistic example of how complex interventions are currently developed and evaluated. The lessons learned from this case study are likely applicable to other teams developing and evaluating complex interventions. Additionally, the availability of quantitative data across three development phases allowed us to illustrate how quantitative analyses can be used to improve complex intervention development. However, because our lessons learned were identified using a single case study, they are not an exhaustive list of factors to consider when developing a complex intervention. Additionally, due to the limitations in the data, all quantitative results in the paper may best be viewed as illustrative examples rather than valid estimates of causal effects. Although we identified areas in which the BetterBirth team’s approach to intervention development could have been improved, the minimally structured multi-phase approach to complex intervention development used by the BetterBirth team is common (see [6, 15, 86, 87] for similar examples). The popularity of this approach may stem from limits on time and resources researchers can dedicate to preliminary studies. Funders may wish to explore more flexible funding mechanisms with longer durations to ensure that researchers have sufficient resources to fully optimize their intervention prior to assessing its effectiveness.

When developing complex interventions, researchers inevitably make difficult decisions that determine the intervention’s ultimate success. We feel that these decisions are more likely to be correct if researchers first develop a robust theory of change that specifies the hypothesized causal relationships between individual variables that are specific to the intervention being developed and the context in which it is being used and then test and refine this theory using quantitative analysis. We acknowledge that initial theories of change, criteria for success, and implementation intensity metrics may undergo substantial changes throughout the complex intervention development process. In addition to quantitative analyses, qualitative research is likely necessary for exploring unanticipated findings, contextualizing results, and generating new hypotheses during formative phases. However, systematically documenting these qualitative contributions and anticipating how they will impact subsequent quantitative analysis will promote learning throughout the intervention development process and give the final version of the complex intervention its best chance of generating meaningful health benefits in a full-scale trial.

## Conclusion

As complex interventions and multi-component implementation packages become more common in health research, identifying strategies for developing and refining these interventions is critical. Theories of change, optimization outcomes, and implementation intensity metrics are generalizable strategies for improving the development and evaluation of complex interventions. By demonstrating the relevance of these strategies and how they can be applied in practice, we hope to encourage the collection and use of data in a way that promotes more effective development and evaluation of complex interventions.

## Data Availability

The datasets used and/or analyzed during the current study are intended primarily as illustrative examples and not intended for secondary data analysis. Data from the CRT phase is available at the Harvard Dataverse repository at https://dataverse.harvard.edu/dataset.xhtml?persistentId=doi:10.7910/DVN/SIQQPG. Remaining data is available from the corresponding author on reasonable request.

## Abbreviations

CRT: Cluster randomized trial
EBP: Essential birth practice
LAGO: Learn-as-You-Go
MOST: Multiphase Optimization Strategy
TOC: Theory of change
